# Who’s Involved? Case Reports on Older Adults’ Alcohol and Tobacco Use in Dutch Residential Care Facilities

**DOI:** 10.1177/10497323231186879

**Published:** 2023-07-10

**Authors:** Lisette de Graaf, Meriam Janssen, Tineke Roelofs, Katrien Luijkx

**Affiliations:** 1 Department of Tranzo, School of Social and Behavioral Sciences, 120694 Tilburg University, Tilburg, The Netherlands; 2 Mijzo, Waalwijk, The Netherlands; 3 Archipel Zorggroep, The Netherlands

**Keywords:** alcohol, alcoholism, tobacco use, long-term care, older people

## Abstract

Residential care facilities (RCFs) provide 24/7 care to older adults with cognitive and/or physical disabilities and aim to provide person-centered care (PCC). Maintaining residents’ autonomy is important to provide PCC, for example, with shared decision-making (SDM). Residents are largely dependent on multiple stakeholders, which could jeopardize their autonomy, especially regarding unhealthy behaviors, such as smoking tobacco or drinking alcohol. This case study explores the dynamics of multiple stakeholders around four RCF residents regarding their alcohol and/or tobacco use. Four RCF residents who smoke tobacco and/or drink alcohol were selected from a previous study, and their (in)formal caregivers were additionally invited to participate. A qualitative research design was chosen, and semi-structured interviews were conducted. The Ethics Review Board from the Tilburg University School of Social and Behavioral Sciences (Reference: RP39) and the executive boards of the two participating organizations granted approval. Narrative portraiture resulted in four case descriptions. Two cases focused mostly on tobacco use, and two cases focused mostly on alcohol use. Multiple stakeholders were involved on different levels: family bought alcohol or cigarettes, and team managers supported care professionals. However, little interaction was found between stakeholders. In these cases, limited interaction between the stakeholders, including the resident, jeopardizes SDM and, in this way, PCC regarding residents’ alcohol and/or tobacco use. SDM on this topic could enhance interaction between all stakeholders involved, which could increase PCC. Finally, the cases indicate a constant struggle between protecting residents from adverse outcomes of alcohol and tobacco use and enhancing their autonomy.

## Background

Residential care facilities (RCFs) aim to provide person-centered care (PCC), which tends to be holistic and based on the beliefs and values of the individual resident (McCormack & McCance, 2021). Moving to an RCF could be necessary for older adults when they become in need of 24/7 care due to cognitive and/or physical disabilities. These older adults spend the final phase of their lives in these facilities. In the Netherlands, the length of stay varies from periods between less than 3 months up to 2 years (Zorginstituut Nederland, 2021). Despite having cognitive disabilities, it is important for RCF residents to make their own decisions. Establishing a shared decision-making process, such as involving RCF residents in day-to-day (care) decisions, is an essential aspect of providing PCC (Daly et al., 2018; Edvardsson et al., 2008). In previous research, it was found that having a sense of choice and volition will enhance residents’ experienced well-being (Kloos et al., 2019).

Due to their physical and/or cognitive disabilities, RCF residents will be inevitably dependent on their (in)formal caregivers to at least some degree (Fazio et al., 2018; Gerritsen et al., 2004), such as when making the choice to smoke tobacco or drink alcohol. Although this seems straightforward, the multiple stakeholders may have opposing perspectives with regard to unhealthy behavior, which could complicate the shared decision-making process on this topic. On the one hand, residents’ dependency could jeopardize their autonomy. Informal and formal caregivers could minimize this potential decrease of autonomy by providing residents with choices about their alcohol and/or tobacco use. On the other hand, (in)formal caregivers feel responsible for the health and safety of all residents (Holmes et al., 2019). From this perspective, they may prioritize safety, health, and the minimizing of potential harm for the individual residents and their environment (Rapaport et al., 2020), which could be contrary to the residents’ wishes in cases of unhealthy behaviors, such as those regarding alcohol and/or tobacco use.

Previous research emphasized the involvement of multiple stakeholders, more specifically the triangle between the resident and formal and informal caregivers, in the maintenance of the autonomy of RCF residents (Boumans et al., 2019; Hoek et al., 2021). Other studies assessed this triangle in home care relationships and found a constant struggle between safety and autonomy, which became more complicated when the needs and wishes of the older adult started to differ from the responsibility and safety issues of (in)formal caregivers (Moermans et al., 2022; Rapaport et al., 2020). Moreover, other studies found that the knowledge, attitudes, and substance use of formal caregivers could also affect their perspectives and behavior toward the alcohol and tobacco use of patients (Bakhshi & While, 2014; Juranić et al., 2017; Pipe et al., 2009). However, to the best of our knowledge, the current field of research does not explore the combined role of multiple stakeholders in RCF residents’ alcohol and/or tobacco use.

This study explores the dynamics of multiple stakeholders around four individual RCF residents who smoke tobacco and/or drink alcohol, with the aim of answering two research questions: (1) How are multiple stakeholders involved in the alcohol and tobacco use of residents living in RCFs? (2) How do the knowledge, attitudes, and personal substance use of these stakeholders affect their behavior toward residents’ use?

## Methods

A qualitative research design was chosen to collect and analyze the data in this study. This design was chosen to gain an understanding of the dynamics of multiple stakeholders around residents’ alcohol and/or tobacco use. Semi-structured interviews were used to gather in-depth information.

### Participants and Research Context

Two Dutch RCFs located in the south of the Netherlands participated in this study. Both organizations participate in the Academic Collaborative Centre (ACC) Older Adults of Tilburg University (Luijkx et al., 2020). Similar to other RCFs in the Netherlands, these organizations provide complex, continuous, and 24/7 long-term residential care for older adults with severe physical and psychogeriatric disabilities (Schols et al., 2004).

Dutch national policies focus on the prevention of smoking tobacco and drinking alcohol, which may affect the care policies in Dutch RCFs. National policies emphasize banning all kinds of tobacco use since July 2023, while policies on alcohol use tend to focus more on preventing problematic alcohol use instead of banning this use altogether (Ministerie van Volksgezondheid, Welzijn & Sport, 2018). In this study, both alcohol and tobacco use are included. Although there are differences in these substances, for example, regarding the impact on the environment, use of both substances challenges care professionals to balance residents’ rights and autonomy with the health and safety of all residents.

For this study, four residents were purposively selected from a sample taken for a previous study (de Graaf et al., 2022) to reach diversity in the cases and the type of care units. The interviews with these four residents were used in the current study. The selected residents all drink alcohol and/or smoke tobacco. Two residents live in a psychogeriatric unit, and two live in a unit in which care is provided for people with severe physical disabilities. In addition to the residents’ interviews, the participating resident was asked which informal caregiver should be invited to participate, mostly a close family member or partner. In Dutch RCFs, there are nurses with a vocational level of education who coordinate the care of individual residents. The coordinating nurses of the participating residents were invited to participate and were asked which other professionals were involved with the resident’s alcohol and/or tobacco use. These professionals were invited, and the participants included elderly care physicians, social workers, and team managers. In these cases, the team managers are responsible for the nursing staff of the units where the participating residents live. In total, 16 care professionals were asked to participate and 13 agreed to participate. Two elderly care physicians and one family member did not respond to the invitation or subsequent reminder.

### Data Collection

Data collection took place from June until December 2020. The semi-structured interviews were based on a topic list (Appendix 1). The duration of the interviews varied between 30 and 60 minutes. One researcher (LG) conducted all the interviews at a conference room of the RCF or at the home of the participant, depending on the preference of the participant. The researcher (LG) works as a psychologist in one of the participating organizations and is experienced in communication with RCF residents. She was not clinically involved with any of the participants. The interviews were audio-recorded and transcribed verbatim.

### Data Analysis

Narrative portraiture was used to analyze the data because this method enabled us to delineate each story, including the story’s context, such as the time and space of events, and how alcohol and tobacco use are conceptualized and experienced by the stakeholders (Rodríguez-Dorans & Jacobs, 2020). All four cases included interviews with the resident and multiple stakeholders. The interview transcripts were independently coded by two researchers, each using an analytic tool from Rodríguez-Dorans and Jacobs (2020) (Table 1). One researcher (LG) coded the transcripts of all cases, and two other researchers (MJ and TR) each coded the transcripts of two cases. Eventually, all transcripts were summarized into four cases based on the study’s analytic tool (Table 1). The perspectives of the residents and multiple stakeholders, such as interactions and relationships between the involved stakeholders, the context, and key events, were compared across these transcripts, which led to case descriptions of the dynamics between a given resident and the stakeholders. The case descriptions were discussed by the researchers to reach consensus on each case. The findings are described in the four case descriptions in the Results section.

**Table 1.** Analytic Tool for Narrative Portraits.^a^

Category	Description	Examples
Character	Who: important characters, relationships between characters	The person him- or herself, experiences involving other people
Time	When: historical context, time in which interview took place	Time periods: weeks, months, days
Space	Where: geographical, political, cultural, social, economic context	Macro: country, city Micro: RCF, apartment Virtual: state of mind
Key events	How/why: connections and relations, interactions, turning points	Links to important decisions, events that change the narrative
Phenomena of interest	How/why: how are the phenomena of interest conceptualized and experienced	Themes of interest, identity
Other	Important subjects or themes that do not fit another category	-

^a^Based on Rodríguez-Dorans and Jacobs (2020).

## Ethical Considerations

The Ethics Review Board from the Tilburg University School of Social and Behavioral Sciences granted approval (Reference: RP39), and the executive boards of the two participating organizations also granted approval. All participants received an information letter and, if they were willing to participate, provided a written informed consent letter prior to enrollment in this study. In the Netherlands, residents living in psychogeriatric units have severe dementia and, due to their cognitive disabilities, are considered legally incapacitated. Therefore, the approval of these residents’ legal representatives was needed and obtained for these residents to participate in the study (Roelofs et al., 2017). When a resident or his or her legal representative agreed to participate, the interview with the resident from the previous study was included in this study.

## Results

This section consists of four case descriptions. Each case starts with an overview of the participants’ characteristics, such as their age, gender, and their own use of alcohol and tobacco. This overview is followed by a description of the dynamics between the resident and multiple stakeholders regarding the resident’s alcohol and/or tobacco use. Moreover, the knowledge and attitudes of the multiple stakeholders toward alcohol and tobacco in general and among RCF residents in particular are described and compared.

### Frank

Frank (fictive name) lives on a unit in which care is provided for residents with mainly physical disabilities. He drinks alcohol occasionally and quit smoking a long time ago. He is happy with his current use. When it comes to smoking tobacco within the RCF, he argues that smoking areas are pleasant for smoking residents but unpleasant for those who do not smoke. Moreover, he describes, “Everyone should decide for themselves to smoke. If someone enjoys it, they should do it. No one else can decide this.” He also accepts alcohol use in others as long as they do not become annoying: “Some people become annoying when drinking alcohol, I don’t need that.” The vocational nurse and the team manager do not know whether Frank drinks alcohol or smokes tobacco nor do they talk with him about this topic.

The vocational nurse and the team manager reflected on their attitudes toward smoking and drinking in general and specifically among RCF residents. Both do not smoke tobacco themselves (Table 2). The vocational nurse argues that people in general society should decide for themselves to smoke or not, although she tries to motivate friends and family who smoke to quit. When it comes to RCF residents, she argues that RCF residents should also decide for themselves to smoke tobacco, if they know the negative health consequences (i.e., a shortened life expectancy and an increase in existing health problems). She describes, “Residents are in the final phase of their lives and, if they enjoy it, I will not motivate them to quit … they should continue smoking … I will discuss why they started smoking and what they have tried to quit, but I will not tell them to quit*.*” According to the team manager, smoking is a dirty habit in general society as well as among RCF residents: “It is sad that residents associate smoking with relaxation.” She argues that residents should be discouraged from smoking as that is the RCF policy.

**Table 2.** Description of Characteristics of “Frank*”.

Role	Age	Gender	Tobacco	Alcoholic consumptions
Frank, resident on a unit providing care for people with severe physical disabilities	91	Male	None	Drinks alcohol occasionally when his son visits him or other specific occasions
Vocational nurse who coordinates his care	22	Female	None	Drinks alcohol occasionally on the weekends at parties; 5–6 glasses of alcohol; sometimes no alcohol for weeks
Team manager	42	Female	None	A daily glass of wine
Son (no response)**	Missing value	Missing value	Missing value	Missing value
Elderly care physician (no response)**	Missing value	Missing value	Missing value	Missing value

*Fictive name.

**Not included in this study due to providing no response to the invitation to participate in the study.

Both the team manager and the vocational nurse drink alcohol themselves (Table 2). They describe that people in general society should decide for themselves to drink alcohol, although it depends on where, when, why, and how much. The team manager expresses a similar attitude when it comes to RCF residents. The vocational nurse also describes that residents should be able to choose for themselves to drink alcohol or not (with a maximum of four or five beers per day). However, she argues that alcohol use in RCF residents is more complicated due to the possible use of medication which could negatively interact with alcohol.

### Margaret

Margaret (fictive name) lives on a psychogeriatric unit. She describes, “My father smoked and I hated that smell. So, I never wanted to smoke*.*” She enjoys drinking alcohol occasionally, and she decides whether to drink or not in her own room. Margaret’s daughter, the vocational nurse who coordinates Margaret’s care, and the team manager are each involved with Margaret’s alcohol use to some degree. Margaret’s daughter buys her alcohol: a bottle of wine and a bottle of eggnog twice a week. At one point, the vocational nurse had noticed that Margaret’s alcohol use had increased, as she had begun to drink in the morning as well. Therefore, the vocational nurse discussed Margaret’s use with her daughter, and, without consulting Margaret, they decided to limit her use by only offering her alcohol in the afternoon. As a consequence, the nurses manage Margaret’s access to alcohol and bring the bottles of eggnog and wine to her room at 2 o’clock in the afternoon, while her daughter describes that Margaret stores these bottles herself. Her daughter also describes that RCF residents could drink alcohol, but it is not actively offered by the nursing staff, while the vocational nurse describes that they offer alcohol actively to residents who drink alcohol and wish to continue their use in the RCF. The team manager does not know that Margaret uses alcohol but describes that she generally supports the nursing staff when problems are experienced with residents’ alcohol and/or tobacco use.

Overall, according to Margaret’s daughter, the vocational nurse, and the team manager, alcohol use by RCF residents should be facilitated. They view alcohol consumption as a normal part of life; it is the residents’ choice if they want to enjoy it. They all drink alcohol themselves as well (Table 3). The team manager and vocational nurse add that residents’ alcohol use should be limited when it negatively affects the resident and/or his or her environment. They do not specify this to Margaret, nor do they further define what “negatively affects” means.

**Table 3.** Description of Characteristics of “Margaret*”.

Role	Age	Gender	Tobacco	Alcoholic consumptions
Margaret, resident psychogeriatric unit	84	Female	None	Self-report: She sometimes has an alcoholic eggnog, but not daily. Her daughter reports Margaret drinks two bottles of wine and two bottles of eggnog per week
Daughter	Missing value	Female	None	Daily, she does not want to tell how much she drinks, but according to her, it is too much
Vocational nurse who coordinates Margaret’s care	27	Female	None	One beer per week (sometimes nonalcoholic) and occasionally a glass of wine in the summer
Team manager	42	Female	None	A daily glass of wine
Elderly care physician (no response)**	Missing value	Missing value	Missing value	Missing value

*Fictive name.

**Not included in this study due to not responding to the invitation to participate in the study.

Although Margaret does not smoke, her daughter, the vocational nurse, and the team manager were asked about their attitudes toward smoking in general and specifically among RCF residents. None of them smoke tobacco themselves (Table 3). The team manager and daughter describe that they see smoking in general as a dirty habit. When it comes to RCF residents, her daughter describes, “When you reach the age of these residents, it should be possible for them to continue smoking and drinking as they already lost so much*.*” However, smoking should be limited to specific smoking areas inside and outside the RCF. This is in line with the view of the vocational nurse. Although she has an aversion to smoking tobacco, she helps residents to smoke. She describes, “If someone already smoked for years, you will maintain their dignity by offering them to continue to smoke within the RCF*.*” In contrast, the team manager describes that the facility has a policy to discourage smoking, and she argues that it is a pity that some residents smoke tobacco. She understands that residents started smoking before the adverse health outcomes were well-known, and residents are addicted to nicotine. Therefore, it will be hard for them to quit smoking.

### John

John (fictive name) lives on a psychogeriatric unit. He mentions, “I cannot drink alcohol … alcohol does not work well with my medication*.*” His sister describes that John used to drink excessively after he lost his job as a truck driver. His sister and the coordinating nurse are currently involved in his alcohol use. Neither the team manager nor the elderly care physician knows about John’s alcohol use. The vocational nurse offers John nonalcoholic beer due to his past alcohol addiction, although occasionally she or her colleagues offer him an alcoholic eggnog. According to her, John does not notice the difference between alcoholic and nonalcoholic drinks. His sister gives him a glass of red wine a few times per year because she describes that he does notice a difference and seems to prefer alcoholic beverages.

All participating stakeholders drink alcohol themselves (Table 4) and argue that RCF residents in general could continue their alcohol use in the RCF. The elderly care physician adds, “I don’t think it is a problem when RCF residents drink alcohol, if this is possible with their medication. I try to limit it to one alcohol consumption per day for the residents due to the interaction with medication*.*” The team manager notices that the nursing staff is hesitant to embrace alcohol use in RCF residents and tends to limit their use. He also notices that families have a great role in the final decision whether a resident is allowed to drink alcohol and describes an example: “If a family does not accept the risk of a resident’s falling due to alcohol use, then that resident’s alcohol use will be limited.”

**Table 4.** Description of Characteristics of “John*”.

Role	Age	Gender	Tobacco	Alcoholic consumptions
John, resident psychogeriatric unit	70	Male	Self-report: 10 cigars per day (according to his sister: 100 per week)	Nonalcoholic beer; once every few months, a glass of red wine with his family
Sister	66	Female	12 cigarettes per day	1–2 glasses of wine per day
Vocational nurse who coordinates his care	Missing value	Female	None	Drinks 2–3 glasses of alcohol per week
Elderly care physician	36	Female	None	Occasionally at parties or in a restaurant
Team manager	Missing value	Male	None	He drinks a few beers during the weekend in the evening at parties or social events

*Fictive name.

John enjoys smoking cigars although he experienced a decrease in his use after he moved to the RCF. All participating stakeholders are involved in his tobacco use and argue that John should be able to smoke because he enjoys smoking and he has smoked his entire life. Except for his sister, none of the participating stakeholders smoke tobacco themselves (Table 4). John’s sister buys him cigars and John smokes independently. According to the vocational nurse, he can continue smoking independently as long as it remains safe for him to do so. In John’s case, safety risks did arise when John smoked indoors during the night and caused a fire in the smoking area. After that, a multidisciplinary team became involved to find an appropriate solution. They decided to close this smoking area during the night, and John accepted that he had to smoke outside.

The stakeholders also describe their attitudes toward smoking among RCF residents in general. John’s sister describes, “Residents should be able to smoke in the RCF. If I ever have to move to an RCF, I hope there is a smoking area where I can smoke. I see how my brother enjoys it.” According to the vocational nurse, if someone has dementia and does not ask to smoke, you should not actively offer a cigar or cigarette. The elderly care physician and team manager argue that residents have often smoked their entire lives and have tried to quit in the past. According to them, if smoking increases the residents’ well-being, it should be facilitated and outweighs the negative health consequences. However, the team manager adds that smoking among RCF residents is complicated because in those settings, care professionals will then be exposed to the second-hand smoke. Therefore, he emphasizes the need to assess each case individually and consider all the factors involved.

### Alice

Alice (fictive name) lives on a unit in which care is provided for residents with mainly physical disabilities. She started smoking when she was ten: “I was ten … smoking secretly with friends*.*” She started drinking alcohol at parties when she was 14. As she got older, her alcohol use increased, and she came to drink six beers per day. She moved to an RCF after having a stroke. She smokes tobacco in the car with her husband, and she self-reports that she drinks three beers per day. She and her husband argue that it should be possible for residents to smoke in a smoking area, but currently smoking is only allowed outside the facility.

All participating stakeholders are involved in Alice’s alcohol use, although there is little interaction between the stakeholders about her use. The vocational nurse, elderly care physician, and team manager drink alcohol themselves (Table 5). Her husband and the social worker do not drink alcohol (Table 5). Her husband quit drinking alcohol after Alice asked him to stop. He buys her cigarettes and beer. He describes, “I wish she would drink less alcohol … I tried so many years to change her alcohol use … I cannot fight over it anymore.” Her elderly care physician and the vocational nurse advised her to quit drinking alcohol, but they describe that it is her own responsibility to change her alcohol use. The vocational nurse explained to Alice that some of her health problems are caused by damage to her liver due to her alcohol use. Despite this intervention, she understands Alice’s use because of everything Alice went through during her life. Alice wishes to continue her alcohol and tobacco use. The team manager set rules specifically with Alice about her alcohol use to limit possible nuisance for other residents and staff. They agreed that Alice’s alcohol use is limited to two beers per day in the restaurant after 15.00 o’clock. Alice and her husband report that she exceeds this limit. According to Alice, she drinks three beers per day, and according to her husband, she drinks six beers per day.

**Table 5.** Description of Characteristics of “Alice*”.

Role	Age	Gender	Tobacco	Alcoholic consumptions
Alice, resident on a unit providing care for people with severe physical disabilities	67	Female	4/5 cigarettes per day	Unclear: 3–6 beers per day. She tells her elderly care physician that she drinks one beer per day; during the interview, she reports that she drinks three beers per day; the team manager reports that they set a rule with Alice to drink a maximum of two beers per day; and her husband reports that she drinks six beers per day
Husband	68	Male	15 cigarettes per day	None
Vocational nurse who coordinates her care	65	Female	None	Drinks during the weekends as well as one glass of wine or beer per night
Elderly care physician	63	Male	None	A few times per year (1–2 cases of alcoholic consumption)
Social worker	53	Female	None	None
Team manager	50	Female	None	Occasionally at parties, once every 1 or 2 months

*Fictive name.

Except for her husband, none of the participating stakeholders smoke tobacco themselves (Table 5). The elderly care physician and social worker know about Alice’s tobacco use. The elderly care physician advised Alice to quit smoking because of its impact on her health problems. In contrast, her social worker describes, “If smoking is one of the things that increases Alice’s well-being, I would help her to continue smoking*.*” None of Alice’s caregivers take further action regarding her alcohol or tobacco use. They describe that they can advise her to quit, but they argue that it is her own responsibility to quit drinking and smoking. The vocational nurse and the team manager do not know whether Alice smokes. They both describe that, in general, nurses help residents to smoke, by bringing them outside to smoke. The team manager adds that nurses should be allowed to refuse to stay near the residents while they are smoking because of the potential health impacts of second-hand tobacco smoke.

## Discussion

This qualitative study explored the dynamics among stakeholders involved with four individual residents living in RCFs with the aim to answer two research questions: (1) How are multiple stakeholders involved in the alcohol and tobacco use of residents living in RCFs? (2) How do the knowledge, attitudes, and personal substance use of these stakeholders affect their behavior toward residents’ use? No results were found on how stakeholders’ own use affected their behavior toward the residents’ alcohol and tobacco use. We found that multiple stakeholders are involved in the alcohol and tobacco use of RCF residents, varying from informal caregivers who buy alcohol, cigars, or cigarettes to a team manager who supports care professionals when they experience problems regarding residents’ alcohol and/or tobacco use. This involvement seems straightforward, as residents are dependent on their (in)formal caregivers to fulfill their needs and wishes (Fazio et al., 2018). However, there is little interaction either between the resident and the stakeholders or among the stakeholders themselves about the residents’ alcohol and/or tobacco use. The stakeholders tended to act from their own perspectives, which seemed to be based on their professional expertise, knowledge, and attitudes toward alcohol and tobacco use in RCF residents. In this study, two cases were mainly focused on alcohol use, and two cases were mainly focused on tobacco use. Moreover, we found that multiple stakeholders did not know about the resident’s alcohol and/or tobacco use. When there is no problem or nuisance experienced by the stakeholders, alcohol and/or tobacco use does not seem to be a topic worth discussing.

In these cases, the limited amount of interaction both between the resident and the stakeholders and among the stakeholders could affect the residents’ alcohol and/or tobacco use. For example, in one case, the resident is limited in the amount of alcohol use she is allowed to drink because she had started drinking in the morning. However, it is unclear who decided that this is a problem or whether the resident was involved in the decision-making process of the chosen solution. One of the requisites of providing PCC is dialogue between those involved in the care of the resident, including the resident (McCormack & McCance, 2021). A way to establish this is with a shared decision-making process (McCormack & McCance, 2021), for example, by involving the resident in day-to-day (care) decisions (Daly et al., 2018). RCF residents, especially residents with dementia, could be overlooked in the decision-making process by care professionals (Mariani et al., 2017). However, these residents are able to participate in the decision-making process, for example, by using simple preference questions (Whitlatch, 2010) and so enhance PCC further. In addition, interaction between all stakeholders was found to be important for this shared decision-making process (Hoek et al., 2021; Mariani et al., 2017). This interaction could be especially important when it comes to shared decision-making regarding residents’ alcohol and tobacco use because this topic could cause a struggle between safety and autonomy as the needs and wishes of the resident may differ from the responsibility and safety issues of the (in)formal caregivers (Moermans et al., 2022; Rapaport et al., 2020). Therefore, the limited amount of interaction between the stakeholders could jeopardize the shared decision-making process and indicates the need to interact with all stakeholders, including the resident.

The cases in this study indicate a difference in the views of stakeholders on alcohol use compared to tobacco use and their views on this use in general society compared to their views regarding the use of RCF residents in particular. Stakeholders view alcohol use more positively than tobacco use and tend to be more tolerant toward RCF residents compared to people in general society. The stakeholders view alcohol as a normal part of daily life and a habit that residents should be able to continue in the RCF. This is similar to the results of Burruss et al. (2015), although several stakeholders describe the risk of alcohol use as interacting negatively with medication. Stakeholders are more divided in their views on tobacco use and emphasize the adverse (health) outcomes of that behavior, which is in line with the focus in general society on the prevention of smoking tobacco (Bell et al., 2009). With both substances, stakeholders tend to argue that residents should be able to continue their habits in the final phase of their lives, although some stakeholders argue that residents should be motivated to quit smoking. Since residents are in the final phase of their lives, stakeholders may argue that smoking tobacco and drinking alcohol are residents’ “last pleasures” which enhance their well-being and thus outweigh the negative (health) outcomes.

Whether the alcohol and/or tobacco use of the resident was limited or facilitated is ambiguous and subjective in these cases. For example, in one case, the sister and vocational nurse acted differently toward the resident’s alcohol use because they differed in their opinion as to whether the resident noticed the difference between alcoholic and nonalcoholic beer or wine. This case indicates that the stakeholders based their decision on their own perspectives. An RCF is a home for residents, and residents should be able to make choices about their lifestyles (Grossi et al., 2021). Family members are visitors to RCF residents and feel responsible to represent residents’ needs and wishes. However, the RCF is also a work environment for care professionals. This results in different and sometimes contrasting interests compared to those of residents, such as competing beliefs regarding the importance of establishing a smoke-free environment versus residents’ freedom to make lifestyle choices (Grossi et al., 2021). Another study found varying attitudes toward alcohol consumption in RCFs that did not have clear policies. The assessments as to whether residents could drink alcohol or not were subjective and were, for example, based on the possibility of an interaction between medication and alcohol, without addressing the residents’ wishes (Johannessen et al., 2021). Providing PCC could be endangered when stakeholders base their decisions on their own perspectives without interacting with each other or the resident, which disempowers the resident.

### Limitations

The aim of this study was to explore the dynamics of stakeholders around four residents regarding their smoking and/or drinking behavior. These cases provided in-depth insights into the dynamics of such a care system, but a small sample, due to the chosen research method, prevents generalization of the results. The nurse who coordinates the care of the resident was asked which formal caregivers were involved in the resident’s alcohol and/or tobacco use. This is another limitation because the residents were not involved in this decision. However, we made this choice because in some cases the residents were not aware who was involved in their use due to their cognitive disabilities. Furthermore, the cases differed in the number of participants and their roles. For example, two cases did not include an elderly care physician and one case did not include an informal caregiver. Therefore, the cases were difficult to compare. However, the richness of the interviews in all cases provided us with valuable insights into the dynamics among stakeholders involved with the care of four individual residents. It showed how these stakeholders are involved in the smoking and/or drinking behavior of residents.

### Future Research

The four cases indicate that providing PCC and maintaining residents’ autonomy could be difficult when it comes to smoking tobacco and drinking alcohol due to the multiple stakeholders involved, their different perspectives and interests, and the limited interaction between them and the resident on this theme. Future research could assess whether a shared decision-making process may enhance PCC in residents’ alcohol and/or tobacco use. A shared decision-making process may enhance PCC because it enhances residents’ involvement and the interaction between the resident and the involved stakeholders. This process could acknowledge the competing interests and limitations within RCFs, not only regarding alcohol and tobacco use but also on other aspects of PCC. Moreover, the perspectives of (in)formal caregivers are important for providing PCC regarding residents’ smoking and/or drinking behavior. Therefore, it could be valuable to further study their attitudes and own use on a larger scale and assess whether these attitudes and own use affect their behavior toward residents’ alcohol and/or tobacco use.

### Practical Implications

Insights from this study could be useful for daily care practice as they can encourage care professionals to be aware of their own attitudes and behavior toward residents’ alcohol and tobacco use. Moreover, this study indicates that there is little interaction on this topic between the stakeholders, including the resident, and not all stakeholders know about the residents’ alcohol and/or tobacco use. The shared decision-making process regarding day-to-day care decisions could be used to enhance the interaction. Using this process is important to provide PCC, especially in cases of unhealthy behaviors where the complexity of providing PCC becomes visible, such as residents’ smoking and/or alcohol drinking behavior.

## Appendix

### Appendix 1: Topic List

**Table:**

*General questions*	How old are you? Where were you born and raised? What do you think when you hear the word “alcohol”? What do you think when you hear the word “tobacco”?
*Smoking tobacco*	
Topic	Example questions
Personal use of tobacco	Do you smoke? Can you tell me more about this? - When do you smoke? - When do you not smoke? - What feeling do you get when you smoke? - How much do you smoke a day? - How have your smoking habits changed over time?
Wishes concerning tobacco	If the choice were up to you, would you choose to smoke? Where are you allowed to smoke in this RCF and how is smoking facilitated or regulated? What do you expect regarding the rules about smoking in this RCF?
Reasons to use or not to use tobacco	Could you tell me why you smoke tobacco or why you don’t smoke tobacco? Were these reasons similar in the past?
Knowledge of the consequences of tobacco	What do you know about the mental or physical consequences of smoking tobacco? - What do you know about the advantages? - What do you know about the disadvantages?
Attitude toward tobacco	What do you think when you smoke tobacco? What do you think when people in your environment smoke tobacco? What do you think when people living in RCFs smoke tobacco?
Smoking behavior of the participating resident	What can you tell about the tobacco use of this resident? - Does he or she smoke? - When does he or she smoke? - With whom does he or she smoke? - What are his or her reasons for smoking? - What do you think of his or her use? - What do you do to facilitate or regulate his or her use?
*Alcohol*	
Topic	Example questions
Personal alcohol use	Do you drink alcohol? Can you tell me more about this? - When do you drink alcohol? - When do you not drink alcohol? - What kind of feeling do you get when you drink alcohol? - How have your drinking habits changed over time?
Wishes concerning alcohol use	If the choice were up to you, would you choose to drink alcohol? How is drinking alcohol regulated or facilitated in this RCF? What do you expect regarding the rules about drinking alcohol in this RCF?
Reasons to drink or not to drink alcohol	Could you tell me why you drink alcohol or why you don’t drink alcohol? Were these reasons similar in the past?
Knowledge of the consequences of alcohol use	What do you know about the mental and physical consequences of drinking alcohol? - What do you know about the advantages? - What do you know about the disadvantages?
Attitudes toward alcohol use	What do you think when you or people in your environment drink alcohol?
Drinking behavior of the participating resident	What can you tell about the alcohol use of this resident? - Does he or she drink alcohol? - When does he or she drink alcohol? - With whom does he or she drink alcohol? - What are his or her reasons for drinking alcohol? - What do you think of his or her use? - What do you do to facilitate or regulate his or her use?
